# Slovenian population data for five new European Standard Set Short tandem repeat loci and SE33 locus

**DOI:** 10.3325/cmj.2014.55.14

**Published:** 2014-02

**Authors:** Irena Zupanič Pajnič, Eva Podovšovnik Axelsson, Jože Balažic

**Affiliations:** 1 Institute of Forensic Medicine, Faculty of Medicine, University of Ljubljana, Ljubljana, Slovenia; 2 Turistica – Faculty of Tourism Studies, University of Primorska, Portorož, Slovenia

## Abstract

**Aim:**

To establish the allele distribution and statistical parameters of forensic interest for the D10S1248, D22S1045, D2S441, D1S1656, D12S391, and SE33 loci in Slovenian population and to compare allele frequencies with those from other populations.

**Methods:**

We analyzed blood and buccal swab samples from 333 unrelated, healthy Slovenian individuals. All samples were genotyped using the AmpFlSTR NGM Kit to obtain the allele frequency data for the loci D10S1248, D22S1045, D2S441, D1S1656, and D12S391. Samples from 113 individuals were also analyzed using the PowerPlex ESX 17 system to obtain the allele frequency data for the SE33 locus. Allele frequencies and statistical parameters of forensic interest were determined and frequency profiles compared between Slovenian and other European Caucasian populations using the Arlequin software, version 3.5.1.3.

**Results:**

The investigated short tandem repeat (STR) loci in Slovenian population had a great discriminating potential with a combined discrimination power of 0.99999998. The highest discrimination power and polymorphism information content were observed for the SE33 locus, followed by loci D1S1656, D12S391, D10S1248, D2S441, and D22S1045. When Slovenian allele frequency distribution was compared with other European populations, deviations were found only for Spanish and Italian population for D2S441 and D12S391.

**Conclusion:**

Slovenian population does not differ significantly from other European populations in terms of allele frequency distributions for the six analyzed STR loci. Based on forensic efficiency values, SE33 may be considered the most informative locus, which makes it especially useful in forensic investigations.

In their recommendations for autosomal short tandem repeat (STR) DNA typing in forensic casework, the European Network of Forensic Institutes (ENFSI) and the European DNA Profiling (EDNAP) Group proposed the use of additional five STRs, three mini-STRs (D2S441, D10S1248, D22S1045), and two highly polymorphic STRs (D1S1656 and D12S391). The increased number of the European Standard Set (ESS) loci resulted in improved discrimination power, sensitivity, and reproducibility for the analysis of minute amounts of DNA (1,2). In the last few years, several commercial STR typing kits have been released, with five new ESS loci. The new kits have been shown to be robust enough to successfully genotype even degraded DNA from old bone material (3-5). These kits also include the AmpFlSTR NGM^TM^ PCR Amplification Kit (Applied Biosystems, Foster City, CA, USA) and the PowerPlex ESX 17 System (Promega, Madison, WI, USA), which we used for our population study. Allele frequencies for autosomal STRs (6,7), Y-chromosomal STRs (8), and mitochondrial DNA (9) were already determined for Slovenian population and the aim of this study was to apply new genetic markers in routine forensic casework to achieve higher evidential value of STR typing and to increase the number of short STR loci, which are better preserved in degraded samples. Some European population studies have already investigated the new ESS loci (D10S1248, D22S1045, D2S441, D1S1656, D12S391) and we compared Slovenian allele frequencies with them (10-17). Beside the analysis of 5 new ESS loci we also analyzed SE33 locus and compared allele frequencies with Austrian (15), Italian (16), German (18), and Spanish population (19).

## Materials and methods

### Population sample and DNA extraction

The population sample consisted of 333 healthy, unrelated people originating from all geographical regions of Slovenia. Samples were taken from routine casework performed at the Institute of Forensic Medicine, University of Ljubljana, in the period from 2010 to 2013, and the research project was approved by the Medical Ethics Committee of the Republic of Slovenia. From paternity trios, we selected only fathers and mothers and from complex kinship analyses only unrelated individuals. We collected buccal smears on sterile cotton swabs and bloodstains on QIAcard FTA^TM^ cards (Qiagen, Hilden, Germany). The DNA was extracted and purified in a Biorobot EZ1 (Qiagen) device using the EZ1 DNA Investigator Card and EZ1 DNA Investigator Kit (Qiagen). Following the manufacturer’s instructions (20), the Biorobot EZ1 was used to obtain genomic DNA from bloodstain samples using the trace protocol and from buccal swab samples using the “tip dance” protocol. The final volume of extracts was 50 µL. The DNA extracts from all buccal swabs and bloodstain samples were quantified using the Quantifiler^TM^ Human DNA Quantification Kit (Applied Biosystems) and the quantity of DNA was determined. The reactions were carried out in a 7500 Real Time PCR System (Applied Biosystems), using the HID Real-Time PCR Analysis Software, version 1.1 (Applied Biosystems) according to the manufacturer’s instructions (21).

### STR typing

DNA typing of autosomal STR loci was performed for all samples using the AmpFlSTR NGM^TM^ PCR Amplification Kit (Applied Biosystems). Additionally, 113 of 333 samples underwent duplicate analysis using the PowerPlex ESX 17 System (Promega) to obtain the data for SE33 locus. We used both kits to increase the posterior probability in complex kinship analyses in cases when the probability for inclusion was too low after typing autosomal STRs using NGM kit. With typing of additional highly polymorphic SE33 locus using PowerPlex ESX 17 it was possible to achieve higher evidential value of autosomal STR typing. We performed a population analysis of 5 new ESS loci including 333 individuals and of the SE33 locus including 113 individuals. The NGM and ESX 17 System contain the same 15 core STR loci, including five new ESS loci, and amelogenin, whereas the ESX 17 System also contains the SE33 locus. The polymerase chain reaction (PCR) amplifications were performed according to the manufacturer’s instructions (22,23). Both reactions were performed using the ABI PRISM 7000 Sequence Detection System (Applied Biosystems). Simultaneously with the population samples, we amplified the positive controls (AmpFlSTR Control DNA 007, Applied Biosystems, and Control DNA 9947A, Promega) and negative PCR controls. The fluorescent-labeled products of the amplification kits were separated with capillary electrophoresis on an automatic ABI PRISM^TM^ 3130 Genetic Analyzer (Applied Biosystems) using the 3130 Performance Optimized Polymer 4 (Applied Biosystems) and the GeneScan-500 LIZ (Applied Biosystems) internal size standard with the NGM kit, and CC5 Internal Lane Standard 500 (Promega) with the ESX 17. The genetic profiles were determined using the Data Collection v. 3.0 and GeneMapper ID v. 3.2 software (Applied Biosystems). Samples with questionable allele calls and with peak imbalance were reanalyzed. For quality control, our laboratory regularly participates in the quality control proficiency testing provided by the GEDNAP group (German DNA profiling group).

### Statistical analysis

We used the Arlequin software, version 3.5.1.3 to determine allele frequencies, observed heterozygosity, expected heterozygosity, probability value of the Hardy-Weinberg equilibrium (HWE) exact test, and standard error (24). The same software was used for the frequency profile comparisons between Slovenian and other European Caucasian populations. For comparison of ESS loci data we used population data from Croatia (10), Czech Republic (11), Belgium (12), Hungary (13), Germany (14), Austria (15), Italy (16), and Spain (17), and for comparison of SE33 locus data we used population data from Austria (15), Italy (16), Germany (18), and Spain (19). Statistical parameters of forensic interest: matching probability, power of discrimination, polymorphism information content, power of exclusion, and typical paternity index were determined for each locus as indicators of their discrimination potential in human identification and paternity analysis applications using the PowerStats, version 12 (Promega, Fitchburg, WI, USA).

## Results

Statistical parameters (probability value of the HWE exact test) for the six STR loci showed no deviations from the HWE, except for the D22S1045 locus (0.0475), which was rejected after Bonferroni correction (25) (Supplementary Table 1)(supplementary table). When we compared allele frequency distribution in our study with other European population studies, we found deviations from the Spanish population only for the STR loci D2S441 (*P* = 0.0497) and D12S391 (*P* = 0.0217) (17). There was also a deviation from Italian population for D12S391 locus (16) (*P* = 0.0471, Table 1).

**Table 1:** Comparison of allele frequencies on 6 short tandem repeat loci between Slovenian population and previously published population data (exact test ± standard error)*

Population/Locus	D10S1248	D1S1656	D22S1045	D2S441	D12S391	SE33
Croatia (10)	0.5957+ -0.0115	0.7215+ -0.0184	0.5405+ -0.0158	0.9057+ -0.0064	0.5539+ -0.0180	
Czech Republic (11)	0.3424+ -0.0189	0.6112+ -0.0130	0.0935+ -0.0102	0.3005+ -0.0247	0.2580+ -0.0129	
Belgium (12)	0.4121+ -0.0210	0.2645+ -0.0290	0.4884+ -0.0265	0.3799+ -0.0357	0.6370+ -0.0217	
Hungary (13)	0.4176+ -0.0229	0.6013+ -0.0239	0.9266+ -0.0030	0.7807+ -0.0149	0.2344+ -0.0198	
Germany (14,18)	0.0992+ -0.0181	0.9149+ -0.0088	0.6479+ -0.0101	0.2858+ -0.0300	0.4895+ -0.0181	0.2262+ -0.0282
Austria (15)	0.3222+ -0.0279	0.3392+ -0.0271	0.5167+ -0.0177	0.5199+ -0.0231	0.4657+ -0.0353	0.1560+ -0.0111
Italy (16)	0.0508+ -0.0120	0.6459+ -0.0163	0.2215+ -0.0099	0.4754+-0.0292	**0.0471+** **-0.0077**	0.6786+ -0.0184
Spain (17,19)	0.6375+ -0.0205	0.3570+ -0.0333	0.0684+ -0.0132	**0.0497+** **-0.0100**	**0.0217+** **-0.0042**	0.4365+ -0.0330

*P value of the exact test of population differentiation. Significant differences (*P* < 0.05) are in bold.

The analyzed STR loci had a combined discrimination power of 0.99999998. The highest discrimination power and polymorphism information content were observed for SE33 locus (0.986 and 0.94, respectively), followed by D1S1656, D12S391, D10S1248, D2S441, and D22S1045. The highest typical paternity index and power of exclusion were observed for the SE33 locus (11.30 and 0.91, respectively) followed by D12S391, D1S1656, D10S1248, D2S441, and D22S1045. The lowest matching probability was calculated for the SE33 locus (0.014), followed by D12S391, D1S1656, D10S1248, D2S441, and D22S1045 (Supplementary Table 1).

## Discussion

The analyzed STR loci in Slovenian population were highly polymorphic, as it was shown previously in other European populations (10-19). SE33 was the most informative locus, which makes it an especially useful locus in forensic investigations. SE33 is a complex tetranucleotide locus and one of the most informative and polymorphic genetic markers used in forensics (18). Among five new ESS loci, D1S1656 and D12S391 were the most discriminating in Slovenian population, as well as in other European populations (10-17).

Allele frequencies for five new ESS loci and SE33 in Slovenian population did not significantly differ from those in Croatia (10), Czech Republic (11), Belgium (12), Hungary (13), Germany (14,18), Austria (15), Italy (16), and Spain (17,19), and allele frequency distributions for the six loci in most European populations were similar. Slovenian allele frequencies differed only from Spanish (17) and Italian population (16), where deviations were observed only for D2S441 and D12S391. Deviations of Slovenian allele frequency distribution from Spanish and Italian population were also observed in previous population data studies (6,7) on the loci TH01, D7S820, and D8S1179. Since the sample size for SE33 locus was small we expected some deviations of allele frequency distribution from Austrian (15), Italian (16), German (18), and Spanish (19) populations, but none were observed. According to Butler (26), a study based on 100-150 participants is generally accepted as appropriate for determining population data.

The obtained data demonstrate that analyzed loci are very useful for forensic purposes due to their shortness and high polymorphism. We recommend that 5 new ESS STR loci are adopted for the analysis of degraded challenging DNA samples to improve the discrimination power of national databases and to facilitate standardization within Europe (1,2). Because of their high informativeness, investigated markers can be applied in complex kinship cases and used for detection of chimerism in patients after hematopoietic stem cell transplantation.
