# Supplementary material for: Slovenian population data for five new European Standard Set Short tandem repeat loci and SE33 locus
Source: Croat Med J. 2014 Feb;55(1):14–8. doi: 10.3325/cmj.2014.55.14 (PMC3944414; doi:10.3325/cmj.2014.55.14)
Supplement: Supplementary Table 1 [file CroatMedJ_55_s004.pdf]

Table 1 **Allele frequencies and statistical parameters of 6 short tandem repeat (STR) loci in Slovenian population sample.***

Allele/Locus	D10S1248	D1S1656	D22S1045	D2S441	D12S391	SE33
6						0.0044
9				0.0015		0.0044
10		0.0015	0.0030	0.1967		
11	0.0015	0.0991	0.1547	0.3273		0.0044
11.3				0.0616		
12	0.0240	0.1472	0.0210	0.0435		0.0089
13	0.2523	0.0736	0.0060	0.0255		0.0089
14	0.3153	0.0766	0.0451	0.3048		0.0310
14.2						0.0044
14.3		0.0030				
15	0.2070	0.1306	0.3679	0.0285	0.0330	0.0398
15.3		0.0526				
16	0.1727	0.1216	0.3003	0.0075	0.0285	0.0310
16.3		0.0481				
17	0.0210	0.0526	0.0841	0.0015	0.0826	0.0797
17.1		0.0030				
17.2						0.0044
17.3		0.1156			0.0090	0.0044
18	0.0060	0.0090	0.0180	0.0015	0.2132	0.0664
18.3		0.0556			0.0135	
19					0.1006	0.0443
19.2						0.0044
19.3		0.0105			0.0075	
20					0.1006	0.0575
21					0.1457	0.0443
21.2						0.0177
22					0.1156	
22.2						0.0398
23					0.0886	
23.2						0.0443
24					0.0390	0.0044
24.2						0.0221
25					0.0210	
25.2						0.0443
26					0.0015	0.0044
26.2						0.0664
27.2						0.0620
28.2						0.1062
29.2						0.0664
30.2						0.0354
31.2						0.0221
32.2						0.0133
33						0.0044

36						0.0044
Population sample	333	333	333	333	333	113
Observed heterozygosity	0.7718	0.8829	0.7417	0.7568	0.8979	0.9558
Expected heterozygosity	0.7643	0.9023	0.7418	0.7552	0.8822	0.9502
HWE exact test (p)	0,6214	0,7673	**0,0475**	0,5032	0,3207	0,1306
Standard error	0,00002	0,00002	0,00001	0,00002	0,00002	0,00002
Matching probability	0.099	0.020	0.120	0.101	0.030	0.014
Power of discrimination	0.901	0.980	0.880	0.899	0.970	0.986
Polymorphism information content	0.72	0.89	0.70	0.72	0.87	0.94
Power of exclusion	0.548	0.761	0.496	0.521	0.791	0.91
Typical paternity index	2.19	4.27	1.94	2.06	4.90	11.30
